# Ethnic Disparities in Police Diversion and Criminalisation for Drug Possession in England: A Study of 32,758 Incidents

**DOI:** 10.1111/dar.70230

**Published:** 2026-08-17

**Authors:** Ashley Mills, Dan Lewer, Guillermo Reyes Pascual, Nadine Hendrie, Paul Quinton, Matthew Bacon, Alex Stevens

**Affiliations:** ^1^ Centre for Health Services Studies, University of Kent Canterbury UK; ^2^ Bradford Institute for Health Research Bradford UK; ^3^ School of Social Sciences University of Kent Canterbury UK; ^4^ College of Policing Durham UK; ^5^ School of Law University of Sheffield Sheffield UK

## Abstract

**Introduction:**

Police‐led drug diversion schemes have expanded across England, yet little is known about whether their use reproduces established ethnic disparities in policing outcomes.

**Methods:**

Using individual‐level data from 32,758 incidents recorded by 15 police forces (covering 38% of England's population), we conducted a cohort study to estimate ethnic differences in the likelihood of: (i) being offered diversion for suspected drug possession; and (ii) receiving a criminal sanction. Mixed‐effects logistic regression models were used to adjust for age, sex, suspected drug type and police‐force area, with missing data addressed via Bayesian chained imputation.

**Results:**

We found substantial disparities. Compared with White suspects, Black suspects had significantly lower odds of receiving an offer of diversion (odds ratio 0.75, 95% confidence interval 0.65–0.87), while Asian suspects had significantly higher odds (odds ratio 1.25, 95% confidence interval 1.09–1.43). For criminalisation, Asian suspects were less likely than White suspects to receive a criminal sanction (odds ratio 0.81, 95% confidence interval 0.74–0.89), while Black suspects showed no statistically significant difference.

**Discussion and Conclusions:**

These findings extend long‐standing evidence of unequal policing, showing that ethnic disparities persist at the earliest decision points of drug law enforcement—even within schemes intended to reduce criminalisation. The results underscore the need for police forces to monitor and remediate disproportionality in diversion decisions. Future work should investigate mechanisms underpinning officers' diversion decisions and assess whether differential access to diversion contributes to cumulative inequalities in police‐community relations and downstream criminal justice outcomes.

## 1 Introduction

There is long‐standing concern about ethnic disparities in the criminal justice system of England and Wales [1, 2, 3]. Landmark reports by Lord Macpherson [4], David Lammy MP [5] and Baroness Casey [6] commissioned by members of the British government have highlighted the presence of institutional racism in various parts of the criminal justice system, such as courts, prisons and probation service, but most notably with Black people's experiences of the police. His Majesty's Inspectorate of Constabulary, Fire and Rescue Services found, for example, that people categorised as Black were three times more likely to be arrested than those categorised as White [7].

A recent innovation in English policing is the introduction of specific schemes that divert people who are caught in possession of illicit drugs away from the criminal justice system and instead provide educational or other supportive interventions^1^ [9]. This is known as police‐led drug diversion, which may reduce criminalisation of people for drug possession [10]. However, these schemes may have different effects for different ethnic groups. Previous analysis has suggested that Black suspects are more likely to receive punitive outcomes at each stage of the policing and prosecution of drug possession [11]. It is possible that this type of inequality will also exist in diversion schemes, and that the probability of being offered diversion as an alternative to criminal justice will differ by ethnic group.

The reasons for racial disparities in police practices are complex [12], but police stereotyping likely plays a pivotal role. Officers frequently associate Black people with drug‐related crime [13, 14], despite evidence showing no higher rates of drug use among Black populations [15]. Such stereotyping is particularly relevant to police‐led drug diversion, as the decision to divert often relies on an officer's subjective assessment of a suspect's “suitability” for support versus punishment.

We estimated ethnic disparities in the use of police diversion and criminalisation among people suspected of possessing illicit drugs in England. The pathway from police contact to diversion includes at least two important milestones. The first is whether the police decide to offer diversion. The second is whether a suspect receives a criminal sanction including a criminal record (sometimes known as the ‘disposal’ or ‘outcome’ of the case).

Although the diversion schemes in our participating forces varied in exact implementation, two major routes to bias are common to them all. Firstly, the front‐line officer is the primary adjudicator in deciding whether an offer of diversion should be made, irrespective of additional downstream mechanisms, so long as the offence itself is eligible [16, 17]. Thus, direct bias based on perceived ethnicity can enter here. And secondly, most schemes require acceptance of responsibility or admission of guilt and all require further co‐operation with the police to proceed. Thus, schemes may indirectly favour groups with more trusting relationships with the police. Because a common alternative to diversion for eligible offences is to proceed with a criminal sanction, diversion and criminalisation covary. A full description of the diversion process is described in detail in the Outcomes section.

Based on our knowledge and understanding of existing research, we tested three hypotheses: (i) police are more likely to offer diversion to some ethnic groups than others; (ii) some ethnic groups are more likely to receive criminal sanctions after being suspected of drug possession; and (iii) people racialised as Black are less likely to be offered diversion and more likely to be criminalised.

Our analysis reveals a stark divergence in outcomes: while Asian individuals are significantly more likely to be offered diversion and less likely to be criminalised relative to White suspects, Black individuals are significantly less likely to be offered diversion and are criminalised at the same rate as White suspects.

## 2 Methods

### 2.1 Study Design

We conducted a cohort study of people suspected of illicit drug possession in England. The exposure was the ethnicity of participants and the outcomes were: (i) offers of diversion; and (ii) criminalisation. We followed a preregistered study protocol [18]. This study is part of a broader research project related to diversion [8].

### 2.2 Data Source

Police forces in the UK are required to adhere to the National Crime Recording Standard as operationalised through the Home Office Crime Recording Rules [19]. When the police suspect an individual of a crime, they are required to record details of the incident including information about the suspect and the outcome of the investigation.

**FIGURE 1:**
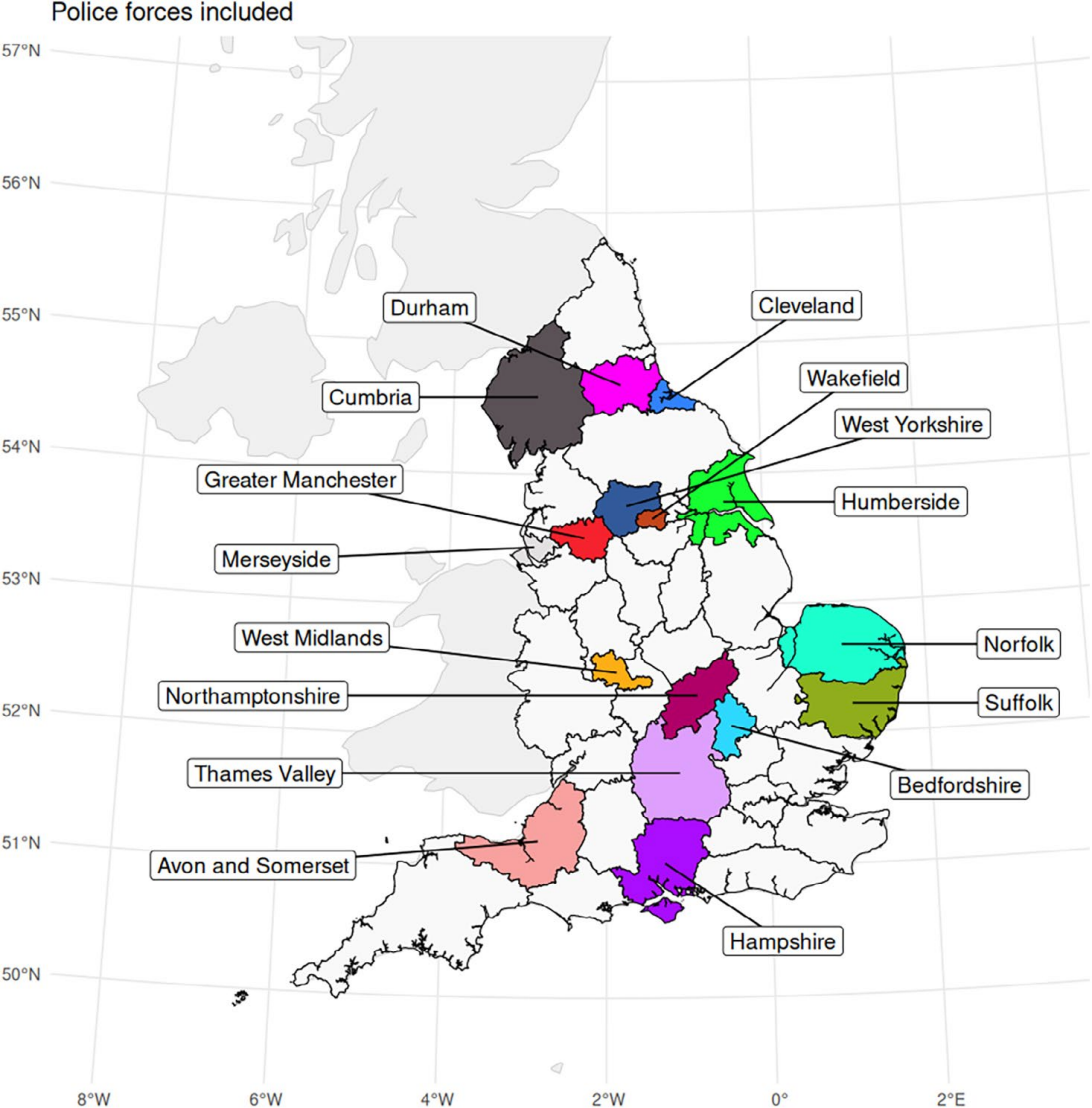
Map of participating police forces.

We invited all police forces in England to share variables about people suspected of illicit drug possession between 1 October 2021 and 30 September 2022. Fifteen police forces participated, covering a population of 21.8 million (38% of the population of England). A map of these police forces is shown in Figure 1.

Note that Wakefield is a precinct of West Yorkshire police. We treated it as an independent force area in our analysis since it offered a diversion scheme which was geographically limited to it.

Each police force provided a table of incidents, such that individuals could have repeated rows in the table if they were suspected of possessing illicit drugs on multiple occasions.

### 2.3 Exposure

The exposure was recorded ethnicity harmonised into five categories: White, Black, Mixed, Asian and other.

Data were derived from two recording modalities: self‐defined ethnicity and officer‐observed identity codes. Because recording practices varied across forces, we prioritised self‐defined ethnicity as the gold standard, falling back to identity codes in its absence. Each scheme was mapped to the five‐category framework to ensure consistency across the combined dataset and to increase statistical power. Full details of the ethnicity mapping methodology are provided in the Supporting Information (see ‘Ethnicity mapping protocol’).

Note, since the term ‘Asian’ has different implications depending on country, in England and Wales Pakistani, Indian and Bangladeshi accounted for 75% of all Asian people recorded in the 2021 census [20] whereas in the US Chinese, Indian, Filipino, Vietnamese and Japanese account for ~75% of all Asian people (Asian alone or in combination) according to 2021 estimates from the US Census Bureau [21].

### 2.4 Outcomes

We studied two outcomes: an offer of diversion and a criminalisation. To understand how diversion operated in practice, we reviewed diversion scheme documentation and operational guidance from participating police forces.

We defined an offer of diversion as either: (i) the police force provided a flag showing that the participant was offered diversion or referred to a diversion scheme; or (ii) the Home Office outcome code was OC22, which is: ‘Diversionary, educational or intervention activity, resulting from the crime report, has been undertaken and it is not in the public interest to take any further action’ [19].

We defined criminalisation as the participant receiving the outcome OC01 or OC01A (‘charged or summons’, meaning the police considered there was enough evidence to charge the participant or proceed to court); or OC03 or OC03A (“caution”, which is a formal warning for a minor offence to which the participant has admitted guilt and forms part of the criminal record). These are the only two home‐office outcome categories that result in criminalisation.

These outcomes are not mutually exclusive: for example, a participant can receive a diversion and, due to non‐compliance, end up being cautioned or charged. Hence each outcome is analysed independently.

The decision process for these outcomes starts when the police have evidence of an eligible offence. In practice this usually means that the police have searched a suspect or vehicle and found drugs. Forces may have different guidelines on what quantity or presentation of drugs meets the threshold for a possession offence and when the circumstances meet the criteria for the more serious offence of possession with intent to supply (which would not be eligible for diversion). For example, the service specification for the West Midlands DIVERT scheme explicitly states that officers must use discretion to determine if the quantity of drugs qualifies as ‘for personal use’, or leads to ‘suspicion of supply’ [22]. Some amount of discretion is evident therefore in determining whether an offence is eligible for diversion, creating the potential for ethnic bias at this stage of the process.

Once a possession offence has been determined, the officer then decides whether diversion is an appropriate disposal. This decision may be influenced by factors such as previous offending history, previous compliance with diversion schemes, the circumstances of the offence, the officer's familiarity and agreement with the diversion scheme, their ‘gut feeling’ about the offender, their personal perceptions of drug use and criminal journeys, and the attitude of the offender and their reaction to the police [16, 17]. The DIVERT scheme service specification states that ‘the officer would then use their discretion to determine what would be the best approach for this individual’. Police officer discretion is therefore crucial in determining whether diversion is used, and who gets diverted.

If diversion is not offered this does not automatically mean that the person will be charged and prosecuted for the offence. The police have a wide range of other disposals that they can use. These include community resolutions and cautions. Many other cases that are not dealt with by diversion are subsequently dropped; often because of ‘evidentiary difficulties’. The distinctive feature of our operationalisation of diversion is that the police forces recorded that they deliberately diverted the suspect to some form of intervention as an alternative to criminalisation.

Once diversion is offered, the suspect must consent to continue, which requires co‐operation with police and diversion providers. In practice this generally involves at least implicit acceptance of responsibility for the possession offence, and some schemes also require an explicit admission of responsibility. For example, the West Midlands DIVERT scheme requires participants to sign a community resolution form, which states “I admit committing this offence and agree to participate in the resolution outlined in Part 1 of this document” [23]. Engagement with the diversion process may therefore depend partly on trust in police and willingness to co‐operate, which may implicitly bias groups with low‐trust in the police.

Finally, schemes vary in whether non‐compliance with the intervention results in re‐evaluation of their case. In the Thames Valley Police diversion scheme, non‐compliant participants are sent back to the case referral team, who then decide which action to take [24]. In contrast, under the West Midlands DIVERT scheme, compliance is effectively voluntary and, while non‐compliance does not reopen the current case, the participant is no longer eligible for diversion in future [23].

**FIGURE 2:**
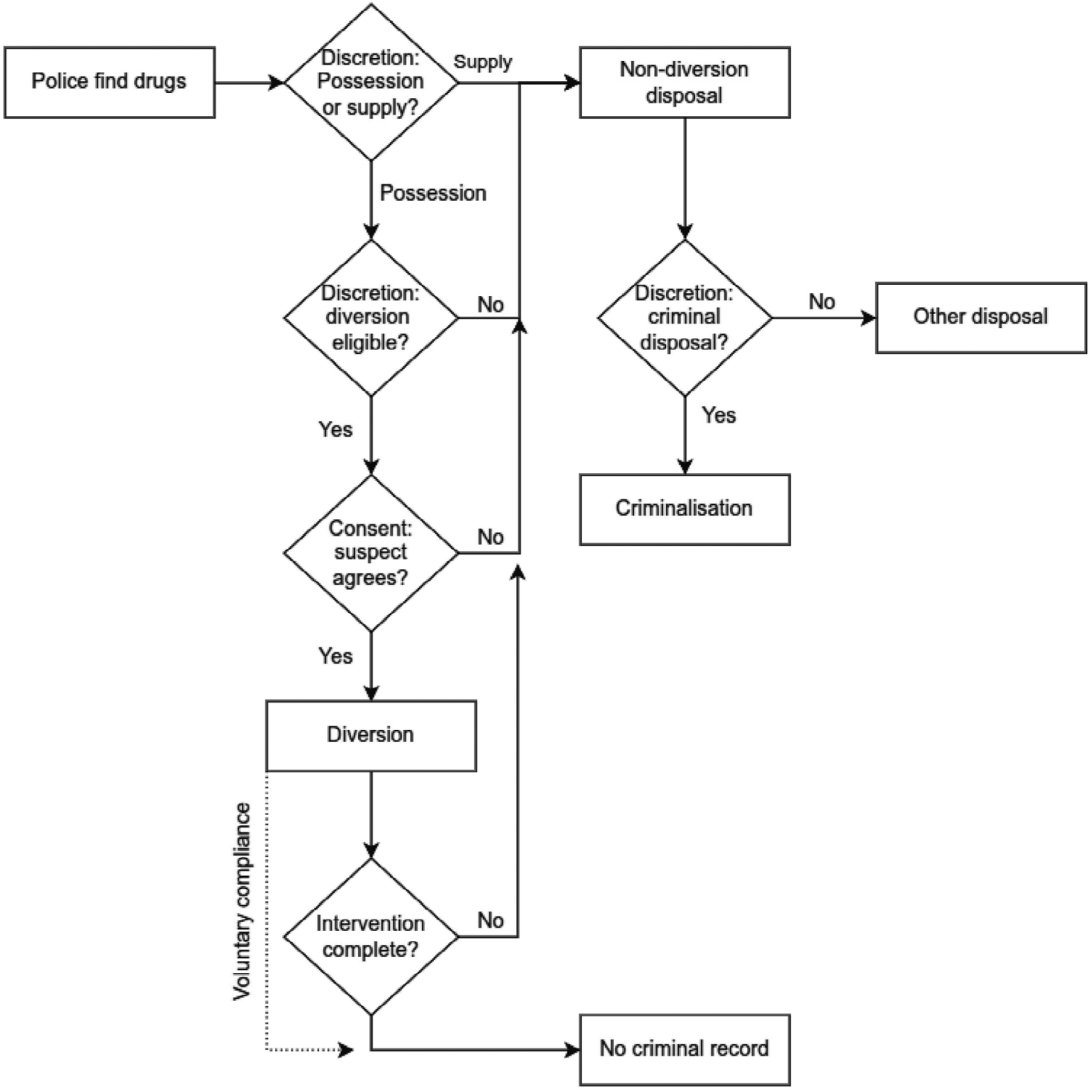
Flowchart explaining how diversion and criminalisation arise from an initial drugs find, showing the points where the process depends on police discretion and suspect compliance.

Figure 2 captures the core diversion pathway, highlighting key decision points at which ethnic disparities could arise through police discretion, suspect consent and responses to non‐compliance.

### 2.5 Confounding Variables

Based on our provisional theory of change [25], we understood diversion and criminalisation as outcomes shaped by individual characteristics, offence circumstances, and local criminal justice context. The broader theory of change describes how diversion may affect later outcomes through reduced criminalisation, reduced stigma and labelling, and improved access to support, but for this analysis its main relevance was in identifying factors likely to influence whether diversion was offered or a criminal sanction was applied. We therefore selected the available covariates that captured these domains: age at the time of the suspected offence, sex, the drug the participant was suspected of possessing, and the police force where the incident occurred.

We did not have data on residential address, so could not include residential area deprivation as a confounder. Prior offending history was not available consistently across all participating forces and was therefore not included in the primary model; we examined its influence in a sensitivity analysis restricted to the forces for which these data were available (see Supporting Information) and found no difference in interpretation.

The data included 20 drug possession offences, indicating specific drugs such as heroin or broader categories such as ‘Other—Class A’. Based on our knowledge of meaningful groupings of illicit drugs and the size of the resulting groups (to ensure that they were not too small), we grouped these drugs into: (i) cannabis; (ii) drugs related to opioid and crack use (heroin, crack cocaine, methadone); (iii) powder cocaine; (iv) other stimulants and hallucinogens (ketamine, MDMA, BZP, amphetamine, methamphetamine, LSD and mephedrone); and (v) other drugs (synthetic cannabinoids, anabolic steroids, khat, GHB, other class A, other class B, other class C and unspecified).

### 2.6 Statistical Analysis

We used a mixed‐effect logistic regression model to estimate the effect of ethnicity on each of our two outcomes. In these models, the dependent variable was a binary indicator showing whether the participant was offered diversion (in the first model) or whether they were criminalised (in the second model). The main independent variable was ethnicity. Other independent variables included to control confounding were age (centred and standardised), age squared (centred and standardised), sex, the group of the drug that the participant was suspected of possessing (see above), and a random intercept for the police force. To estimate crude associations between ethnicity and the outcomes, we also fitted: (i) unadjusted models (i.e., including the dependent variable and ethnicity as the only independent variable); and (ii) models that were only adjusted for age, sex and the drug, excluding the random intercept for the police force. The fully adjusted model equation is shown below:

$$\begin{array}{c} \log\left[\frac{P\left(\text{Diverted}=1\right)}{1-P\left(\text{Diverted}=1\right)}\right]=\beta_{0}+\gamma_{0j}+\beta_{1}\left(\mathrm{Age}\right)+\beta_{2}\left(\mathrm{Ag}e^{2}\right)+\beta_{3}\left(\mathrm{sex}\right)\\ \;+\beta_{4}\left(\text{Ethnicity}\right)+\beta_{5}\left(\text{Offence}\right) \end{array}$$

where

$$\gamma_{0j}∼N\left(0,\sigma_{\text{ForceName}}^{2}\right)$$

Data were missing for ethnicity (3727/32,758 incidents; 11.4%), sex (175/32,758; 0.5%) and home office outcomes (475/32,758; 1.5%). We excluded the 175 incidents with missing sex.

We used a chained Bayesian model to impute missing ethnicity and Home Office outcome codes because procedures using off‐the‐shelf Gibbs sampling and bootstrap algorithms produced implausible distributions in the missing observations. Missingness appeared to be associated with observed characteristics of cases and differences in recording practices across police forces. By conditioning on these observed variables, the Bayesian imputation model was designed to capture observed factors likely to be associated with missingness: as with standard multiple imputation approaches, this assumes that missingness is at random conditional on the variables included in the imputation model.

First, we imputed missing ethnicity observations. In this model, the dependent variable was ethnicity, and independent variables were the diversion outcome, sex, age, age squared, whether the participant accessed drug or alcohol treatment in the past 5 years, the group of the drug they were suspected of possessing, whether the incident involved multiple offences, and a random intercept for the police force. We generated 20 imputations from the resulting posterior distribution. We then used each imputed dataset as a basis for imputing the home office outcome code.

For Home Office outcome, we used a similar model as the one described above, except that the home office outcome code was the dependent variable and the ethnicity was included as an independent variable. From each posterior we generated a single imputation of missing outcome codes. This two‐stage chained imputation resulted in 20 complete datasets. We repeated our analysis using each dataset and used Rubin's Rules to pool the results.

All analysis was done using R version 4.5.2 [26], with the Bayesian imputation procedure implemented using the ‘brms’ R package version 2.22 [27].

## 3 Results

A total of 32,758 incidents were included in the analysis. These incidents occurred among 30,234 individuals, with 7% of individuals having more than one incident, and 1% having more than two. The median age of participants at the time of the incident was 28 (interquartile range 22–36); 87.1% were male; and 70.6% were suspected of possessing cannabis. Table 1 shows baseline sociodemographic and criminological characteristics of this cohort as well as the frequency of the two outcomes.

**TABLE 1:** Characteristics of the sample univariate descriptive, including ethnicity, covariates, outcomes (reported diversion and criminal sanction by outcome 1 and 3 vs. all others).

Variable	Category	*N*	%
Sex	Male	28,542	87.1
	Female	4043	12.3
	Missing	173	0.5
Age at last birthday	Median [IQR]	28 [22–36]	
	[18,25]	13,694	41.8
	[25,35]	10,110	30.9
	[35,45]	5778	17.6
	[45,55]	2489	7.6
	[55,65]	608	1.9
	[65,100]	79	0.2
Ethnicity	White	22,076	67.4
	Asian or Asian British	3394	10.4
	Black, African, Caribbean or Black British	2222	6.8
	Mixed or multiple ethnic groups	926	2.8
	Other ethnic groups	413	1.3
	Missing	3727	11.4
Drug of suspected possession	Cannabis	23,117	70.6
	Powder cocaine	3952	12.1
	Other	2995	9.1
	Opiates and crack cocaine	1764	5.4
	Other stimulants and hallucinogens	930	2.8
Offered diversion	Yes	8614	26.3
	No	24,144	73.7
Received a criminal record as a result of this offence	Yes	23,147	70.7
	No	9136	27.9
	Unknown	475	1.5

Abbreviation: IQR, interquartile range.

The largest ethnic group was ‘White’, accounting for 67.4% of participants. The next largest were ‘Asian or Asian British’ (10.4%), ‘Black, African, Caribbean or Black British’ (6.8%), ‘Mixed or multiple ethnic groups’ (2.8%) and ‘Other ethnic groups’ (1.3%).

Table 2 compares the proportions of ethnic categories between forces, in addition to the overall and 2021 census proportions for England. The distribution of ethnicity of participants varied substantially across police forces, with the proportion of participants from ethnicities other than “White” ranging from 3% in Durham to 57% in the West Midlands.

**TABLE 2:** Proportions of non‐missing ethnicity categories, by participating police force, all participating forces combined, and the 2021 census proportions for England.

Source	% White	% Asian	% Black	% Mixed	% Other
Greater Manchester	70.49	15.07	10.69	2.13	1.61
West Midlands	43.37	29.58	18.47	6.88	1.71
Merseyside	92.25	0.99	2.73	2.35	1.68
Hampshire	89.20	4.16	5.91	0.18	0.54
Thames Valley	73.08	11.21	8.57	5.81	1.32
Bedfordshire	56.06	22.89	16.49	3.39	1.16
Humberside	94.57	2.77	2.43	0.00	0.23
West Yorks (Excluding Wakefield)	59.51	26.94	7.37	5.08	1.09
Cumbria	96.38	2.66	0.72	0.24	0.00
Wakefield	88.60	4.79	3.47	2.48	0.66
Avon and Somerset	83.80	2.32	8.13	2.12	3.62
Northamptonshire	81.66	4.89	9.26	2.97	1.22
Cleveland	89.59	3.98	2.19	2.96	1.29
Durham	97.52	0.62	0.83	0.62	0.41
Norfolk	89.77	2.27	7.95	0.00	0.00
Suffolk	86.67	1.67	11.67	0.00	0.00
Total police	76.04	11.69	7.65	3.19	1.42
Census 2021	81.05	9.61	4.22	2.96	2.18

### 3.1 Diversion

In the primary analysis, Asian or Asian British participants were more likely to be offered diversion when compared to White participants (odds ratio [OR] 1.25, 95% confidence interval [CI] 1.09–1.43), while Black, African, Caribbean or Black British participants were less likely (OR 0.75, 95% CI 0.65–0.87). We did not find evidence that other ethnic groups had different odds of being offered diversion when compared to White participants (Table 3). In models 1 and 2, which did not include a random intercept for the police force, the association between Asian or Asian British ethnicity and diversion was reversed.

**TABLE 3:** Odds ratios estimating the association between ethnicity and being offered diversionary interventions among people suspected of possessing illicit drugs in 2021 and 2022, with 95% confidence intervals.

Ethnicity	Model 1 (unadjusted)	Model 2 (adjusted for age, sex, drug)	Model 3 primary analysis (adjusted for age, sex, drug, police force)
White	1 (ref)	1 (ref)	1 (ref)
Asian or Asian British	0.76 (0.70, 0.84)	0.69 (0.63, 0.76)	1.25 (1.09, 1.43)
Black, African, Caribbean or Black British	0.75 (0.67, 0.83)	0.70 (0.62, 0.78)	0.75 (0.65, 0.87)
Mixed or multiple ethnic groups	1.02 (0.88, 1.19)	0.94 (0.81, 1.09)	0.86 (0.72, 1.04)
Other ethnic groups	1.28 (1.02, 1.59)	1.18 (0.94, 1.47)	0.97 (0.74, 1.26)

The Supporting Information contains sensitivity analyses for the diversion and criminalisation outcomes comprising: (i) complete case analysis; (ii) the addition of prior offending and drug treatment history covariates; (iii) person‐level clustering; (iv) an alternative drug classification; and (v) an alternative ethnicity mapping that prioritises officer perception. In all cases the interpretation of the fully adjusted models does not change.

### 3.2 Criminalisation

In the primary analysis, Asian or Asian British participants were less likely to be criminalised when compared to White participants (OR 0.81, 95% CI 0.74–0.89). Participants in “other ethnic groups” were also less likely to be criminalised (OR 0.77, 95% CI 0.60–0.99). We did not find evidence of differences in the probability of criminalisation between participants of White ethnicities and those of Black, African, Caribbean or Black British ethnicities, or those of mixed or multiple ethnic groups (Table 4).

**TABLE 4:** Odds ratios estimating the association between ethnicity and criminalisation among people suspected of possessing illicit drugs in 2021 and 2022, with 95% confidence intervals.

Ethnicity	Model 1	Model 2 (adjusted for age, sex, drug)	Model 3—primary analysis (adjusted for age, sex, drug, police force)
White	1 (ref)	1 (ref)	1 (ref)
Asian or Asian British	0.66 (0.61, 0.71)	0.77 (0.70, 0.84)	0.81 (0.74, 0.89)
Black, African, Caribbean or Black British	0.83 (0.76, 0.92)	0.96 (0.87, 1.06)	1.08 (0.97, 1.20)
Mixed or multiple ethnic groups	0.77 (0.66, 0.90)	0.87 (0.74, 1.02)	0.98 (0.83, 1.15)
Other ethnic groups	0.59 (0.46, 0.74)	0.65 (0.51, 0.83)	0.77 (0.60, 0.99)

## 4 Discussion

In this individual‐level study of 32,758 drug possession incidents, we found evidence of ethnic disparities in diversion offers. Black suspects were less likely than White suspects to be offered diversion, while Asian suspects were more likely to be offered diversion and less likely to be criminalised. These estimates are conditional on recorded police contact for suspected drug possession and therefore describe disparities after this point in the enforcement pathway rather than disparities in police contact.

This study makes three main contributions. First, to our knowledge, it is the first study to use individual‐level police incident data to examine ethnic disparities in diversion and criminalisation among people suspected of drug possession.

Second, our denominator is directly relevant for the estimand. Our outcome estimates are denominated by the recorded incident‐level ethnicity proportions of actual drug possession suspects, rather than their proportions in residential census populations. This is important because census denominators can obscure differences in baseline police presence, differential police contact likelihood, neighbourhood and other contextual factors that may influence who comes in contact with the police [28], [29].

Previous work estimating stop‐and‐search likelihood with respect to ethnicity has also moved beyond crude residential census denominators [30]. They decomposed search likelihood among White, Asian and Black suspects into differences in area‐level patrolling (over/under policing) and differences within contact events (officer bias). In this case the area‐level patrolling denominator did use census ethnicities but these were weighted by actual patrol patterns. Whereas the officer bias denominator was derived from recorded contact event counts for each officer and thus aligns with their estimand. While they estimated both contact likelihood and post‐contact search likelihood using a single force and were able to account for officer characteristics, they did not control for individual suspect characteristics beyond recorded ethnicity. In contrast, we estimated post‐contact diversion and criminalisation likelihoods across multiple forces, and we adjusted for suspect drug, age, sex, in addition to ethnicity.

Third, our exposure was ethnicity mapped to five broad categories, rather than a binary White/minority grouping. This is important because the findings differed in direction for Black and Asian suspects. A previous study that estimated ethnic disparities in the likelihood of receiving a custodial sentence at Crown Courts in England and Wales found sentencing disparities that were not explained by area deprivation [31]. They however collapsed ethnicity into the binary White/minority. Our findings suggest that such aggregation may attenuate, exaggerate or even reverse associations between ethnic groups.

The lower likelihood of diversion among Black suspects is consistent with longstanding evidence of ethnic disparities across criminal justice pathways, including stop and search, arrest and sentencing [5, 7, 15]. Our findings extend this evidence to an earlier and less studied decision point: whether people suspected of drug possession are offered diversion rather than processed through other criminal justice routes. This suggests that schemes intended to reduce criminalisation may still reproduce unequal access at the point of referral.

We did not find evidence that Black suspects were more likely than White suspects to be criminalised. Criminalisation is not a simple complement of diversion; incidents may end through other disposals such as no further action or evidential difficulties. It is possible that there are discrepancies in the use of outcomes that are not directly criminalising, but which differ in the burdens they place on either suspects or the criminal justice system. Future work should examine these pathways since they may reveal less obvious locations of unequal treatment.

Asian and Asian British suspects were more likely than White suspects to be offered diversion and less likely to be criminalised after accounting for police force, age, sex and suspected drug type. This finding differed from the unadjusted analysis, in which Asian suspects appeared less likely to be offered diversion. The unadjusted pattern reflects the interaction between force demographics and the provision of diversion; diversion forces had a smaller share of Asian interactions. In forces with diversion schemes Asian suspects accounted for 1747 incidents (5.9%), whereas in forces without diversion schemes Asian suspects accounted for 3568 incidents (13.1%). Force‐adjustment addresses this imbalance by comparing outcomes within force context, avoiding the conflation of between‐force differences in ethnicity with differences in diversion provision.

The reasons for the adjusted association are uncertain. It may reflect unmeasured differences in case mix, offence context, suspect admission or cooperation, local eligibility rules, recording practice, or differential discretionary decision‐making by officers. Our data cannot distinguish between these explanations. Police forces should therefore treat this finding as a prompt for local audit and explanation, rather than as evidence that the underlying mechanism is understood.

The study had several limitations. First, the study period overlapped with the later stages of the COVID‐19 pandemic and the transition out of restrictions. This may have affected overall levels of police contact, diversion provision, and case processing. However, all ethnic groups were observed over the same period, and the primary analysis included police force to account for local differences in policing context and diversion implementation. Residual bias remains possible if pandemic‐related disruption affected ethnic groups differently within forces.

Second, the analysis was conditional on recorded police contact for suspected drug possession. The stop‐and‐search study discussed earlier [30] found that Asian over‐representation in stop‐and‐search was explained primarily by over‐patrolling of Asian areas (a pre‐contact factor), whereas Black over‐representation was explained by a combination of over‐patrolling and officer bias (a contact factor). Since our data comprises people already recorded by the police as suspected of drug possession, it cannot explain discrepancies in how people entered the cohort, only differences conditional on recorded police contact. The differences we observe between Asian and Black treatment might be explained by different pathways through selection into contact and decision making after contact, but this study does not have the data to estimate the former.

Third, we harmonised ethnicity to five broad categories to address heterogeneous recording practices between forces and to increase statistical power. While this is an improvement on a White/minority binary, as it allowed us to observe differences between Black and Asian people, pooling may still obscure important within‐category differences in outcome.

Fourth, while we adjusted our results for age, sex, suspected drug type and police force, and conducted sensitivity analyses to include historical offending and drug treatment counts (See Supporting Information), unobserved individual factors such as drug quantity, suspect attitude towards the police and socioeconomic status may act as residual confounders. We also did not adjust for officer characteristics since we did not have this data.

These findings have implications for the equitable use of police‐led diversion schemes and highlight that the possibility for unfair treatment can occur at many different stages of the criminal justice process, even within schemes designed to reduce criminalisation.

## 5 Conclusion

We found that the odds of being offered police‐led diversion for suspected drug possession differed by recorded ethnicity. Black suspects had lower odds of being offered diversion than White suspects, while suspects recorded in Asian categories had higher odds of being offered diversion and lower odds of criminalisation. These findings show that ethnic disparities in diversion are not adequately captured by a simple White/minority comparison.

Police‐led diversion schemes are intended to reduce criminalisation, but our findings suggest that access to diversion itself may differ by ethnicity. The lower likelihood of diversion among Black suspects is particularly concerning because it aligns with longstanding evidence that Black people experience disadvantage across multiple stages of the criminal justice pathway. The finding for Asian suspects also requires explanation because it indicates a different pattern of post‐contact outcomes that would be obscured by aggregating minority categories.

Following the Lammy Review's principle that ethnic disparities in the criminal justice system should be explained or reformed [5], police forces should routinely monitor the use of diversion to ensure that it is offered fairly and consistently among eligible suspects. Where differences in access to diversion cannot be explained by differences in eligibility, local context, or other observed factors, schemes should be reviewed and reformed. Reviews should look beyond formal eligibility criteria and consider the roles that officer discretion, suspect trust in police, and communication play in brokering a successful diversionary outcome across all stages of the process.

## Author Contributions

Ashley Mills conducted the statistical analysis and edited the manuscript. Dan Lewer led the statistical design. All authors contributed to drafting, critically reviewing and revising the manuscript, and approved the final version.

## Funding

This work was supported by the Cabinet Office Evaluation Accelerator Fund, UK Government and National Institute for Health and Care Research.

## Conflicts of Interest

The authors declare no conflicts of interest.

## Supporting information


**Table S1:** Balance between observed ethnicity in the complete case data, with the average percentage in the data with the imputed cases included. Averaged over 20 imputations.
**Table S2:** Odds ratios estimating the association between ethnicity and being offered diversion among people suspected of possessing illicit drugs in 2021 and 2022, with 95% confidence intervals. Results are shown for complete case analysis where incidents with missing sex, ethnicity, or outcome (excluding those with a positive police flag for diversion) are dropped.
**Table S3:** Odds ratios estimating the association between ethnicity and criminalisation among people suspected of possessing illicit drugs in 2021 and 2022, with 95% confidence intervals. Results shown for complete case analysis where incidents with missing sex, ethnicity or home office outcome are dropped. Table S4 and Table S5 show the results for the new models for the diversion and criminalisation outcomes respectively.
**Table S4:** Odds ratios estimating the association between ethnicity and being offered diversionary interventions among people suspected of possessing illicit drugs in 2021 and 2022, with 95% confidence intervals where in addition to the primary covariates, models 2 and 3 were adjusted for prior offending and drug treatment service usage.
**Table S5:** Odds ratios estimating the association between ethnicity and criminalisation among people suspected of possessing illicit drugs in 2021 and 2022, with 95% confidence intervals where in addition to the primary covariates, models 2 and 3 were adjusted for prior offending and drug treatment service usage.
**Table S6:** Odds ratios estimating the association between ethnicity and being offered diversionary interventions among people suspected of possessing illicit drugs in 2021 and 2022, with 95% confidence intervals where in addition to the primary covariates, models 2 and 3 were adjusted for prior offending and drug treatment service usage, plus a person level clustering.
**Table S7:** Odds ratios estimating the association between ethnicity and criminalisation among people suspected of possessing illicit drugs in 2021 and 2022, with 95% confidence intervals where in addition to the primary covariates, models 2 and 3 were adjusted for prior offending and drug treatment service usage, plus person level clustering.
**Table S8:** Odds ratios estimating the association between ethnicity and being offered diversionary interventions among people suspected of possessing illicit drugs in 2021 and 2022, with 95% confidence intervals where in addition to the primary covariates, models 2 and 3 were adjusted for prior offending and drug treatment service usage. And finally in this model, the drug possession category was simplified to cannabis/not‐cannabis.
**Table S9:** Odds ratios estimating the association between ethnicity and criminalisation among people suspected of possessing illicit drugs in 2021 and 2022, with 95% confidence intervals where in addition to the primary covariates, models 2 and 3 were adjusted for prior offending and drug treatment service usage. And finally in this model, the drug possession category was simplified to cannabis/not‐cannabis.
**Table S10:** Mismatches between officer perceived ethnicity and self perceived ethnicity in our cohort. The number of each type of mismatch ordered by frequency is shown along with the percentage and cumulative percentage of the total of those mismatched.
**Table S11:** Odds ratios estimating the association between ethnicity and being offered diversionary interventions among people suspected of possessing illicit drugs in 2021 and 2022, with 95% confidence intervals where in addition to the primary covariates, models 2 and 3 were adjusted for prior offending and drug treatment service usage, plus a person level clustering. In this case when both the police and suspect recorded ethnicity, conflicts were resolved towards the officer perceived ethnicity.
**Table S12:** Odds ratios estimating the association between ethnicity and criminalisation among people suspected of possessing illicit drugs in 2021 and 2022, with 95% confidence intervals where in addition to the primary covariates, models 2 and 3 were adjusted for prior offending and drug treatment service usage. In this case when both the police and suspect recorded ethnicity, conflicts were resolved towards the officer perceived ethnicity.
**Table S13:** Marginal and conditional goodness‐of‐fit and discrimination metrics for the complete‐case diversion and criminalisation models. *R* ^2^ metrics were calculated using Nakagawa's method for generalised linear mixed‐effects models. Marginal *R* ^2^ represents the variance explained by the fixed‐effect covariates. Conditional *R* ^2^ represents the variance explained by the full model, including both fixed effects and the police‐force random intercept. Conditional AUC measures discrimination while incorporating the random intercept.
**Table S14:** Mapping between identity codes and ethnicity used in the statistical model.
**Table S15:** Mapping between self‐defined ethnicity codes (2011 census codes) codes and ethnicity used in the statistical model.
**Table S16:** Police forces in England and Wales and their participation in this research.

## Data Availability

Data sharing not applicable to this article as no datasets were generated or analysed during the current study.

## Endnotes

^1^
There is evidence of informal use of discretion to divert people away from prosecution for drug possession which goes back to 1934 [8].

## References

[1] R. Hood , Race and Sentencing: A Study in the Crown Court: A Report for the Commission for Racial Equality (Clarendon Press, 1992).

[2] B. Bowling and C. Phillips , Racism, Crime and Justice (Pearson Education, 2002).

[3] H. S. Bhui , Race and Criminal Justice (SAGE, 2008).

[4] Home Office , “The Stephen Lawrence Inquiry,” (1999), https://www.gov.uk/government/publications/the‐stephen‐lawrence‐inquiry.

[5] D. Lammy , “The Lammy Review: Final Report,” (2017), https://www.gov.uk/government/organisations/lammy‐review.

[6] L. Casey , The Casey Review: A Review Into the Standards of Behaviour and Internal Culture of the Metropolitan Police Service (Home Office, 2023).

[7] HMICFRS , “Race and Policing: An Inspection of Race Disparity in Police Criminal Justice Decision‐Making,” (2023), https://hmicfrs.justiceinspectorates.gov.uk/publication‐html/inspection‐of‐race‐disparity‐in‐police‐criminal‐justice‐decision‐making/.

[8] A. Stevens , N. Hendrie , M. Bacon , et al., “Evaluating police drug diversion in England: protocol for a realist evaluation,” Health Justice 11, no. 1 (2023): 46, 10.1186/s40352-023-00249-2. 37968494 PMC10652635

[9] M. Bacon , “From Criminalisation to Harm Reduction? The Forms and Functions of Police Drug Diversion in England and Wales,” Policing and Society 34, no. 3 (2024): 105–123, 10.1080/10439463.2023.2267729.

[10] A. Stevens , C. E. Hughes , S. Hulme , and R. Cassidy , “Depenalization, Diversion and Decriminalization: A Realist Review and Programme Theory of Alternatives to Criminalization for Simple Drug Possession,” European Journal of Criminology 19, no. 1 (2022): 29–54, 10.1177/1477370819887514.

[11] A. Stevens , Drugs, Crime and Public Health: The Political Economy of Drug Policy (Routledge‐Cavendish, 2011).

[12] P. Quinton , “From Estimating to Explaining and Eliminating Ethnic Disproportionality in Stop and Search,” Political Quarterly 95, no. 3 (2024): 489–497, 10.1111/1467-923X.13433.

[13] K. Murji , “White Lines: Culture, “Race” and Drugs,” in Drugs: Culture, Controls and Everyday Life, ed. N. South (Sage, 1999), 49–65, http://www.uk.sagepub.com/booksProdDesc.nav?prodId=Book205798.

[14] P. Quinton , “The Formation of Suspicions: Police Stop and Search Practices in England and Wales,” Policing and Society 21, no. 4 (2011): 357–368, 10.1080/10439463.2011.610193.

[15] M. Shiner , Z. Carre , R. Delsol , and N. Eastwood , “The Colour of Injustice: “Race”, Drugs and Law Enforcement in England and Wales,” http://www.stop‐watch.org/.

[16] A. Stevens , W. Agnew‐Pauley , M. Bacon , et al., “Cascading Constraint and Subsidiary Discretion: Perspectives on Police Discretion From Police‐Led Drug Diversion and Stop and Search in England,” British Journal of Criminology 66, no. 2 (2026): 448–466, 10.1093/bjc/azaf050.

[17] M. Bacon , “Implementation Fidelity Matters: Insights From a Realist Evaluation of Police Drug Diversion Schemes in England,” Politica y Sociedad 0, no. 0 (2026): 1–24, 10.1080/10439463.2026.2651196.

[18] D. Lewer , “The Effect of Police Diversion Schemes on Offending and Health for People Suspected of Drug‐Related Offences Analysis Protocol. V2.0,” (2024), https://discovery.ucl.ac.uk/id/eprint/10196619/1/pdd_quantitative_protocol_v2.0.pdf.

[19] Home Office , “Home Office Crime Recording Rules for Frontline Officers and Staff,” (2025), https://www.gov.uk/government/publications/counting‐rules‐for‐recorded‐crime.

[20] Office for National Statistics , “Ethnic Group, England and Wales: Census 2021,” (2026), https://www.ons.gov.uk/peoplepopulationandcommunity/culturalidentity/ethnicity/bulletins/ethnicgroupenglandandwales/census2021.

[21] United States Census Bureau , “B02018: Asian Alone or in any Combination by Selected Groups,” (2026), https://data.census.gov/table/ACSDT1Y2021.B02018?q=Asian.

[22] West Midlands Police and Crime Commissioner , “Service Specification for a Pre‐Arrest Drug Diversion Scheme Statement of Requirement,” (2020), https://www.westmidlands‐pcc.gov.uk/wp‐content/uploads/2020/07/Pre‐Arrest‐Drug‐Diversion‐Spec.docx?x47511.

[23] N. Hendrie , “The West Midlands Police Drug Diversion Scheme DIVERT: Descriptive Manual,” (2023), https://kar.kent.ac.uk/101852/.

[24] N. Hendrie , “The Thames Valley Police Drug Diversion Scheme. TVP: Descriptive Manual,” (2023), https://kar.kent.ac.uk/101846/.

[25] A. Stevens and H. Glasspoole‐Bird , Theory of Change of Police Drug Diversion: A Revised Programme Theory (University of Kent, 2023).

[26] The R Core Team , “R: A Language and Environment for Statistical Computing,” (2025), https://cran.r‐project.org/doc/manuals/r‐release/fullrefman.pdf.

[27] P.‐C. Bürkner , “Brms: An R Package for Bayesian Multilevel Models Using Stan,” Journal of Statistical Software 80, no. 1 (2017): 1–28, 10.18637/jss.v080.i01.

[28] J. H. Ratcliffe and S. S. Hyland , “Police Stops and naïve Denominators,” Crime Science 14, no. 1 (2025): 10, 10.1186/s40163-025-00252-y.

[29] P. A. J. Waddington , K. Stenson , and D. Don , “In Proportion: Race, and Police Stop and Search,” British Journal of Criminology 44, no. 6 (2004): 889–914, 10.1093/bjc/azh042.

[30] L. Vomfell and N. Stewart , “Officer Bias, Over‐Patrolling and Ethnic Disparities in Stop and Search,” Nature Human Behaviour 5, no. 5 (2021): 566–575, 10.1038/s41562-020-01029-w. 33462473

[31] J. Pina‐Sánchez , A. Morales , E. Guilfoyle , A. Veiga , and S. Geneletti , “Testing the Interrelationship Between Area Deprivation and Ethnic Disparities in Sentencing,” Analyses of Social Issues and Public Policy 25, no. 1 (2025): e12446, 10.1111/asap.12446.

