# Supplementary material for: Ethnic Disparities in Police Diversion and Criminalisation for Drug Possession in England: A Study of 32,758 Incidents
Source: Drug Alcohol Rev. 2026 Aug 17;45(6):e70230. doi: 10.1111/dar.70230 (PMC13481990; doi:10.1111/dar.70230)
Supplement: Supplementary file 1 [file DAR-45-0-s001.docx]

# Supplementary Material

## Balance of imputed data

We compared the ethnicity distribution in the observed complete-case data with the average distribution across the 20 imputed complete datasets. Differences were small (<0.6 percentage points for all categories), indicating that the imputation procedure preserved the overall composition of the sample while accounting for observed associations between ethnicity, diversion pathway, and police force. Table S1 shows the comparison.

**Table S1** - Balance between observed ethnicity in the complete case data, with the average percentage in the data with the imputed cases included. Averaged over 20 imputations.

| EthnicityCoarse | Observed % | Mean observed with missing imputed % |
| --- | --- | --- |
| WHITE | 76.04 | 75.47 |
| ASIAN | 11.69 | 11.94 |
| BLACK | 7.65 | 7.84 |
| MIXED | 3.19 | 3.33 |
| OTHER | 1.42 | 1.42 |

## Complete case analysis

A sensitivity analysis was conducted using only incidents with complete observations (N=28,520 for the diversion outcome, and N=28,485 for the criminalisation outcome). The modelled odds for the diversion and criminalisation outcomes are shown in Table S2 and Table S3 respectively. Note that the number of observations are slightly more for the diversion outcome, because those with a police flag for diversion are considered complete, even if the home office outcome code was missing.

**Table S2**: Odds ratios estimating the association between ethnicity and being offered diversion among people suspected of possessing illicit drugs in 2021 and 2022, with 95% confidence intervals. Results are shown for complete case analysis where incidents with missing sex, ethnicity, or outcome (excluding those with a positive police flag for diversion) are dropped.

| **Ethnicity** | **Model 1  (unadjusted)** | **Model 2  (adjusted for age,  sex, drug, prior offending, drug treatment history)** | **Model 3 - primary  analysis  (adjusted for age,  sex, drug, prior offending, drug treatment history, force)** |
| --- | --- | --- | --- |
| White | 1 (ref) | 1 (ref) | 1 (ref) |
| Asian or Asian British | 0.58 (0.53, 0.63) | 0.53 (0.48, 0.58) | 1.28 (1.11, 1.47) |
| Black, African, Caribbean or Black British | 0.64 (0.57, 0.71) | 0.60 (0.54, 0.67) | 0.75 (0.66, 0.87) |
| Mixed or multiple ethnic groups | 0.93 (0.80, 1.08) | 0.86 (0.74, 1.01) | 0.87 (0.72, 1.05) |
| Other ethnic groups | 1.23 (1.00, 1.51) | 1.14 (0.93, 1.41) | 0.97 (0.75, 1.25) |

**Table S3**: Odds ratios estimating the association between ethnicity and criminalisation among people suspected of possessing illicit drugs in 2021 and 2022, with 95% confidence intervals. Results shown for complete case analysis where incidents with missing sex, ethnicity or home office outcome are dropped.

| **Ethnicity** | **Model 1** | **Model 2  (adjusted for age, sex, drug)** | **Model 3 - primary analysis  (adjusted for age, sex, drug, police force)** |
| --- | --- | --- | --- |
| White | 1 (ref) | 1 (ref) | 1 (ref) |
| Asian or Asian British | 0.69 (0.63, 0.75) | 0.80 (0.73, 0.87) | 0.80 (0.73, 0.88) |
| Black, African, Caribbean or Black British | 0.87 (0.79, 0.96) | 1.00 (0.90, 1.10) | 1.09 (0.98, 1.21) |
| Mixed or multiple ethnic groups | 0.80 (0.69, 0.93) | 0.89 (0.77, 1.04) | 0.97 (0.83, 1.14) |
| Other ethnic groups | 0.58 (0.46, 0.74) | 0.64 (0.50, 0.82) | 0.75 (0.59, 0.97) |

In both cases the direction and magnitude of the results are very similar to the fully imputed analysis, and the interpretation of results does not differ.

## Controlling for offending history and drug treatment service usage

As a sensitivity test we fitted models that took account of individual level prior offending and drug treatment service usage. Data was not available for Norfolk and Suffolk for these measures due to unresolved differences regarding the Data Sharing Agreement (DSA), thus results are presented for the remaining 13 forces. For ease of interpretation, only complete observations have been used.

PNC offence records were obtained from the Ministry of Justice by deterministically matching our police index data on PNC number, first name, last name, date of birth, and sex. Police force area was used to confirm matches. The offence records of each participant were re-indexed to their police incidents and for each incident we calculated historical offending counts relative to each incident in the last 5 years for four offence categories: possession offences, other drugs offences, theft offences, and violent offences.

For each participant, we extracted their drug treatment records via the Department of Health and Social Care (DHSC) from NDTMS using a deterministic join on DOB, Sex, Initials, and police force area. For further details see [1]. The drug treatment records of each participant were re-indexed to their police incidents and for each incident we calculated whether the participant was treated for drugs in the last 5 years as a binary variable.

We then added the historical five-year offending count for each of the four offending categories and binary historical five-year drug treatment status to the model formula and refitted the complete case model for the 13 forces for which this data were available.

Table S4 and Table S5 show the results for the new models for the diversion and criminalisation outcomes respectively.

**Table S4**: Odds ratios estimating the association between ethnicity and being offered diversionary interventions among people suspected of possessing illicit drugs in 2021 and 2022, with 95% confidence intervals where in addition to the primary covariates, models 2 and 3 were adjusted for prior offending and drug treatment service usage.

| **Ethnicity** | **Model 1  (unadjusted)** | **Model 2  (adjusted for age,  sex, drug treatment history, offending)** | **Model 3 - primary  analysis  (adjusted for age,  sex, drug, offending, drug treatment history, force)** |
| --- | --- | --- | --- |
| White | 1 (ref) | 1 (ref) | 1 (ref) |
| Asian or Asian British | 0.57 (0.52, 0.62) | 0.51 (0.46, 0.56) | 1.22 (1.06, 1.41) |
| Black, African, Caribbean or Black British | 0.64 (0.57, 0.71) | 0.59 (0.53, 0.66) | 0.78 (0.68, 0.90) |
| Mixed or multiple ethnic groups | 0.93 (0.80, 1.08) | 0.89 (0.76, 1.03) | 0.92 (0.76, 1.12) |
| Other ethnic groups | 1.24 (1.00, 1.52) | 1.15 (0.93, 1.42) | 0.97 (0.74, 1.26) |

**Table S5**: Odds ratios estimating the association between ethnicity and criminalisation among people suspected of possessing illicit drugs in 2021 and 2022, with 95% confidence intervals where in addition to the primary covariates, models 2 and 3 were adjusted for prior offending and drug treatment service usage.

| **Ethnicity** | **Model 1  (unadjusted)** | **Model 2  (adjusted for age,  sex, drug, offending, drug treatment history)** | **Model 3 - primary  analysis  (adjusted for age,  sex, drug, offending, drug treatment history, force)** |
| --- | --- | --- | --- |
| White | 1 (ref) | 1 (ref) | 1 (ref) |
| Asian or Asian British | 0.70 (0.64, 0.76) | 0.84 (0.76, 0.92) | 0.80 (0.72, 0.88) |
| Black, African, Caribbean or Black British | 0.87 (0.78, 0.96) | 0.98 (0.88, 1.09) | 1.03 (0.92, 1.15) |
| Mixed or multiple ethnic groups | 0.81 (0.70, 0.94) | 0.85 (0.72, 1.00) | 0.90 (0.76, 1.07) |
| Other ethnic groups | 0.59 (0.46, 0.75) | 0.61 (0.47, 0.80) | 0.71 (0.54, 0.93) |

The results show that including historical offending and drug treatment history do not change the interpretation of results.

## Person-level clustering

As a sensitivity analysis person-level clustering was added to the above model, the estimated odds ratios for the diversion and criminalisation outcomes are shown in Table S6 and Table S7.

**Table S6**: Odds ratios estimating the association between ethnicity and being offered diversionary interventions among people suspected of possessing illicit drugs in 2021 and 2022, with 95% confidence intervals where in addition to the primary covariates, models 2 and 3 were adjusted for prior offending and drug treatment service usage, plus a person level clustering.

| **Ethnicity** | **Model 1  (unadjusted)** | **Model 2  (adjusted for age,  sex, drug, offending, drug treatment history, person level clustering)** | **Model 3 - primary  analysis  (adjusted for age,  sex, drug, offending, drug treatment history, person level clustering, force)** |
| --- | --- | --- | --- |
| White | 1 (ref) | 1 (ref) | 1 (ref) |
| Asian or Asian British | 0.57 (0.52, 0.62) | 0.48 (0.44, 0.53) | 1.23 (1.06, 1.43) |
| Black, African, Caribbean or Black British | 0.64 (0.57, 0.71) | 0.51 (0.51, 0.65) | 0.77 (0.67, 0.90) |
| Mixed or multiple ethnic groups | 0.93 (0.80, 1.08) | 0.89 (0.75, 1.04) | 0.92 (0.75, 1.13) |
| Other ethnic groups | 1.24 (1.00, 1.52) | 1.17 (0.93, 1.47) | 0.97 (0.73, 1.29) |

**Table S7**: Odds ratios estimating the association between ethnicity and criminalisation among people suspected of possessing illicit drugs in 2021 and 2022, with 95% confidence intervals where in addition to the primary covariates, models 2 and 3 were adjusted for prior offending and drug treatment service usage, plus person level clustering.

| **Ethnicity** | **Model 1  (unadjusted)** | **Model 2  (adjusted for age,  sex, drug, drug treatment history, offending)** | **Model 3 - primary  analysis  (adjusted for age,  sex, drug, drug treatment history, offending, force)** |
| --- | --- | --- | --- |
| White | 1 (ref) | 1 (ref) | 1 (ref) |
| Asian or Asian British | 0.70 (0.64, 0.76) | 0.83 (0.75, 0.91) | 0.79 (0.72, 0.88) |
| Black, African, Caribbean or Black British | 0.87 (0.78, 0.96) | 0.98 (0.88, 1.09) | 1.03 (0.92, 1.15) |
| Mixed or multiple ethnic groups | 0.81 (0.70, 0.94) | 0.84 (0.71, 0.99) | 0.90 (0.76, 1.06) |
| Other ethnic groups | 0.59 (0.46, 0.75) | 0.60 (0.45, 0.78) | 0.70 (0.53, 0.93) |

The addition of person-level clustering to account for non-independence does not change the interpretation of results.

## Alternative drug category encoding

As a sensitivity analysis a different drug categorisation was employed along with the offending and drug-treatment history controlled model. Instead of having five drug categories, we reduced the drug categories to either Cannabis or Not-Cannabis. The estimated odds ratios for the diversion and criminalisation outcomes are shown in Table S8 and Table S9.

**Table S8**: Odds ratios estimating the association between ethnicity and being offered diversionary interventions among people suspected of possessing illicit drugs in 2021 and 2022, with 95% confidence intervals where in addition to the primary covariates, models 2 and 3 were adjusted for prior offending and drug treatment service usage. And finally in this model, the drug possession category was simplified to cannabis / not-cannabis.

| **Ethnicity** | **Model 1  (unadjusted)** | **Model 2  (adjusted for age,  sex, drug (cannabis or not), drug treatment history, offending)** | **Model 3 - primary  analysis  (adjusted for age,  sex, drug (cannabis or not), drug treatment history, offending, force)** |
| --- | --- | --- | --- |
| White | 1 (ref) | 1 (ref) | 1 (ref) |
| Asian or Asian British | 0.57 (0.52, 0.62) | 0.50 (0.46, 0.55) | 1.22 (1.06, 1.41) |
| Black, African, Caribbean or Black British | 0.64 (0.57, 0.71) | 0.59 (0.52, 0.66) | 0.78 (0.68, 0.90) |
| Mixed or multiple ethnic groups | 0.93 (0.80, 1.08) | 0.89 (0.77, 1.04) | 0.93 (0.76, 1.12) |
| Other ethnic groups | 1.24 (1.00, 1.52) | 1.14 (0.92, 1.40) | 0.96 (0.73, 1.26) |

**Table S9**: Odds ratios estimating the association between ethnicity and criminalisation among people suspected of possessing illicit drugs in 2021 and 2022, with 95% confidence intervals where in addition to the primary covariates, models 2 and 3 were adjusted for prior offending and drug treatment service usage. And finally in this model, the drug possession category was simplified to cannabis / not-cannabis.

| **Ethnicity** | **Model 1  (unadjusted)** | **Model 2  (adjusted for age,  sex, drug (cannabis or not), drug treatment history, offending)** | **Model 3 - primary  analysis  (adjusted for age,  sex, drug (cannabis or not), drug treatment history, offending, force)** |
| --- | --- | --- | --- |
| White | 1 (ref) | 1 (ref) | 1 (ref) |
| Asian or Asian British | 0.70 (0.64, 0.76) | 0.83 (0.75, 0.91) | 0.79 (0.72, 0.88) |
| Black, African, Caribbean or Black British | 0.87 (0.78, 0.96) | 0.96 (0.86, 1.07) | 1.02 (0.91, 1.14) |
| Mixed or multiple ethnic groups | 0.81 (0.70, 0.94) | 0.85 (0.72, 1.00) | 0.90 (0.76, 1.06) |
| Other ethnic groups | 0.59 (0.46, 0.75) | 0.61 (0.47, 0.79) | 0.71 (0.54, 0.93) |

The substitution of our five level drug possession category with a simple Cannabis/Not-Cannabis classification does not materially change the results.

## Officer vs Self defined ethnicity

In our data, 11,561 incidents (35.30%) had both officer and self perceived ethnicity specified. In 677 cases (2.07% of the total sample, 5.86% of those with both types of ethnicity specified) these produced different ethnicity mappings when reduced to the common coarse categories. Table S10 quantifies the nature of these mismatches.

**Table S10** - Mismatches between officer perceived ethnicity and self perceived ethnicity in our cohort. The number of each type of mismatch ordered by frequency is shown along with the percentage and cumulative percentage of the total of those mismatched.

| Officer perceived ethnicity | Self perceived ethnicity | N | % | Cumulative % |
| --- | --- | --- | --- | --- |
| BLACK | MIXED | 241 | 35.6 | 35.6 |
| WHITE | MIXED | 86 | 12.7 | 48.3 |
| ASIAN | MIXED | 73 | 10.8 | 59.1 |
| OTHER | ASIAN | 38 | 5.6 | 64.7 |
| ASIAN | WHITE | 33 | 4.9 | 69.6 |
| ASIAN | OTHER | 33 | 4.9 | 74.4 |
| WHITE | OTHER | 28 | 4.1 | 78.6 |
| WHITE | ASIAN | 26 | 3.8 | 82.4 |
| BLACK | WHITE | 20 | 3.0 | 85.4 |
| OTHER | MIXED | 18 | 2.7 | 88.0 |
| WHITE | BLACK | 18 | 2.7 | 90.7 |
| BLACK | ASIAN | 15 | 2.2 | 92.9 |
| OTHER | WHITE | 14 | 2.1 | 95.0 |
| OTHER | BLACK | 13 | 1.9 | 96.9 |
| ASIAN | BLACK | 11 | 1.6 | 98.5 |
| BLACK | OTHER | 10 | 1.5 | 100.0 |

Approximately 59% of the mismatches are where an individual perceives themselves as Mixed but the officer perceives them of being either White, Black, or Asian. Notably, 38 cases (5.6% of all mismatches) involved disagreement between White and Black classifications, with officers perceiving the individual as White when they self-identified as Black, or vice versa.

Since we estimate diversion and criminalisation likelihood as a function of ethnicity, and the outcome is mediated in part through officer discretion, it could be argued that officer perceived ethnicity is more relevant than self-determined ethnicity in assessing the association. For this reason we conducted a sensitivity test where the ambiguous cases above were resolved in favour of the officer perceived ethnicity instead of the self perceived ethnicity.

For consistency with previous sensitivity analyses in supplementary, we have included criminal history and prior drug treatment covariates, which means the analysis does not include Norfolk and Suffolk for reasons explained above. The results of this sensitivity analysis for the diversion and criminalisation outcomes are shown in Table S11 and Table S12 respectively.

**Table S11** - Odds ratios estimating the association between ethnicity and being offered diversionary interventions among people suspected of possessing illicit drugs in 2021 and 2022, with 95% confidence intervals where in addition to the primary covariates, models 2 and 3 were adjusted for prior offending and drug treatment service usage, plus a person level clustering. In this case when both the police and suspect recorded ethnicity, conflicts were resolved towards the officer perceived ethnicity.

| **Ethnicity**  **(ties resolved in favour of officer perceived ethnicity)** | **Model 1  (unadjusted)** | **Model 2  (adjusted for age,  sex, drug, offending, drug treatment history)** | **Model 3 - primary  analysis  (adjusted for age,  sex, drug, offending, g, drug treatment history, force)** |
| --- | --- | --- | --- |
| White | 1 (ref) | 1 (ref) | 1 (ref) |
| Asian or Asian British | 0.56 (0.51, 0.61) | 0.52 (0.47, 0.57) | 1.24 (1.08, 1.43) |
| Black, African, Caribbean or Black British | 0.57 (0.52, 0.64) | 0.54 (0.49, 0.60) | 0.74 (0.65, 0.85) |
| Mixed or multiple ethnic groups | 1.94 (1.63, 2.31) | 1.77 (1.49, 2.11) | 0.91 (0.74, 1.11) |
| Other ethnic groups | 1.19 (0.97, 1.47) | 1.14 (0.92, 1.41) | 1.01 (0.77, 1.32) |

**Table S12** - Odds ratios estimating the association between ethnicity and criminalisation among people suspected of possessing illicit drugs in 2021 and 2022, with 95% confidence intervals where in addition to the primary covariates, models 2 and 3 were adjusted for prior offending and drug treatment service usage. In this case when both the police and suspect recorded ethnicity, conflicts were resolved towards the officer perceived ethnicity.

| **Ethnicity**  **(ties resolved in favour of officer perceived ethnicity)** | **Model 1**  **(unadjusted)** | **Model 2  (adjusted for age, sex, drug, offending, drug treatment history)** | **Model 3 - primary analysis  (adjusted for age, sex, drug, offending history, drug treatment history police force)** |
| --- | --- | --- | --- |
| White | 1 (ref) | 1 (ref) | 1 (ref) |
| Asian or Asian British | 0.70 (0.65, 0.76) | 0.81 (0.75, 0.89) | 0.82 (0.74, 0.90) |
| Black, African, Caribbean or Black British | 0.88 (0.80, 0.97) | 1.00 (0.90, 1.10) | 1.06 (0.96, 1.17) |
| Mixed or multiple ethnic groups | 0.69 (0.56, 0.84) | 0.79 (0.64, 0.97) | 1.01 (0.82, 1.26) |
| Other ethnic groups | 0.65 (0.51, 0.82) | 0.72 (0.56, 0.92) | 0.83 (0.65, 1.06) |

Both tables show fully adjusted results which do not differ materially from those in the main analysis. The only notable differences are in unadjusted analysis for the mixed category, which is the category most changed by resolution of contradictory ethnicities to the police perceived category.

We also performed the sensitivity check not-including the additional covariates but including Norfolk and Suffolk, and found no material differences to the results shown.

## Goodness of fit and discrimination metrics

To provide descriptive information on model adequacy and discrimination, we calculated Nakagawa’s marginal and conditional pseudo-R² and conditional AUC for the complete-case mixed-effects logistic regression models. We report these for the complete-case models rather than pooling across imputed datasets, as these diagnostics are supplementary and the complete-case analyses produced substantively similar effect estimates to the primary analyses. Table S13 reports these estimates.

**Table S13** - Marginal and conditional goodness-of-fit and discrimination metrics for the complete-case diversion and criminalisation models. Note: R² metrics were calculated using Nakagawa’s method for generalized linear mixed-effects models. Marginal R² represents the variance explained by the fixed-effect covariates. Conditional R² represents the variance explained by the full model, including both fixed effects and the police-force random intercept. Conditional AUC measures discrimination while incorporating the random intercept.

| Model | Marginal R^2^  (Fixed effects) | Conditional R^2^  (Total model) | Conditional AUC  (Discrimination) |
| --- | --- | --- | --- |
| Complete case diversion model  (Adjusted for age, sex, ethnicity, drug treatment history, offending history, and force-level clustering) | 0.030 | 0.786 | 0.907 |
| Complete case criminalisation model  (Adjusted for age, sex, ethnicity, drug treatment history, offending history, and force-level clustering) | 0.185 | 0.312 | 0.776 |

These diagnostics indicate that the sensitivity models captured meaningful variation in both outcomes. For diversion, the large difference between marginal R² (0.030) and conditional R² (0.786), together with the conditional AUC of 0.907, reflects substantial between-force variation in diversion availability, implementation, and recording. For criminalisation, the smaller difference between marginal and conditional R² suggests less force-level variation. These metrics are reported descriptively; the models were specified to estimate ethnic differences rather than optimise prediction.

## Ethnicity mapping protocol

Police in the UK use two different coding schemes to record ethnicity [2]:

1. Identity Codes (IC): originally invented for radio use, sometimes called Pheonix coding or 6+1 refers to 6 ethnicities plus "Unknown" (not-stated treated equivalently). Police officers use visual assessment to determine ethnicity using IC coding. This is the primary point-of-contact assessed ethnicity operationally used by the police. See Table S14.
2. Self Defined Ethnicity (SDE): a Home Office coding scheme equivalent to the 2011 census categories called 18+1 which refers to 18 distinct ethnicities plus "Not Stated". The field is filled in when suspects have contact with the police and are "given the opportunity" to state their self-perceived ethnicity. See Table S15.

It is not possible to directly map IC to SDE or vice-versa since the categories in each do not map one-to-one. We can look at an example for each direction. Firstly, going from IC to SDE: the IC category "IC1 - WHITE – NORTH EUROPEAN" is a meta-category and we do not know which specific SDE category a given instance refers to. Secondly, going from SDE to IC, if someone belongs to the SDE category "W9 - ANY OTHER WHITE BACKGROUND" we cannot tell if they are "IC1 - WHITE NORTH EUROPEAN" or "IC2 - WHITE SOUTH EUROPEAN".

Both coding systems do however map onto the coarse ONS Census categories: White, Black, Mixed, Asian, and Other, although no code in the IC system maps onto Mixed. This mapping was performed independently for the IC and SDE columns when each was present. A final coarse ethnicity was obtained by taking SDE as the preference and using IC in its absence.

**Table S14**: Mapping between IC codes and ethnicity used in the statistical model

| **IC Code** | **Model Ethnicity** |
| --- | --- |
| IC1 - WHITE – NORTH EUROPEAN | White |
| IC2 - WHITE – SOUTH EUROPEAN | White |
| IC3 - BLACK | Black |
| IC4 - ASIAN | Asian |
| IC5 - CHINESE, JAPANESE, OR OTHER SOUTH EAST ASIAN | Asian |
| IC6 - ARABIC OR NORTH AFRICAN | Other |
| IC9 - UNKNOWN | NA |

**Table S15**: Mapping between SDE codes (2011 census codes) codes and ethnicity used in the statistical model

| **2011 Census fine** | **2011 Census coarse** | **Model Ethnicity** |
| --- | --- | --- |
| A1 - INDIAN | Asian or Asian British | Asian |
| A2 - PAKISTANI |  |  |
| A3 - BANGLADESHI |  |  |
| A4 - CHINESE |  |  |
| A9 - ANY OTHER ASIAN BACKGROUND |  |  |
|  |  |  |
| B1 - CARIBBEAN | Black, Black British, Caribbean or African | Black |
| B2 - AFRICAN |  |  |
| B9 - ANY OTHER BLACK BACKGROUND |  |  |
|  |  |  |
| M1 - WHITE & BLACK CARIBBEAN | Mixed or multiple ethnic groups | Mixed |
| M2 - WHITE & BLACK AFRICAN |  |  |
| M3 - WHITE & ASIAN |  |  |
| M9 - ANY OTHER MIXED BACKGROUND |  |  |
|  |  |  |
| W1 - BRITISH | White | White |
| W2 - IRISH |  |  |
| W3 - GYPSY OR IRISH TRAVELLER |  |  |
| W9 - ANY OTHER WHITE BACKGROUND |  |  |
|  |  |  |
| O2 - ARAB | Other ethnic group | Other |
| O9 - ANY OTHER ETHNIC GROUP |  |  |
|  |  |  |
| NS - NOT STATED | NA | NA |

During this process we also filled in rows that had ethnicities that were partially missing, that is for cases where an individual has multiple offence entries and ethnicity is specified for at least one offence (N=284, 0.9% of the cohort, N=424 of the original incident pool)]. The algorithm to fill in the partially missing entries in such cases, along with the number and % of the incidents addressed by each step is shown below:

1. If only one ethnicity is specified, fill in the missing values with this and finish (290, 52.8%).

2. If one SDE forms a majority, fill in the missing values with this and finish (69, 24.3%).

3. If one IC forms a majority, fill in the missing values with this and finish (28, 9.9%).

4. Take the most recent incident for which an ethnicity was specified, and if the ethnicity is unique then fill in the missing values with this and finish (32, 11.3%).

5. When multiple ethnicities are specified for the same date, choose one at random, fill in the missing values with this and finish (5, 1.8%).

Percentages have been included to illustrate that the majority of the time, partially missing cases were resolved unambiguously. This process resolves all of the cases where we have partial information.

## Participant police force statistics

**Table S16** - Police forces in England and Wales and their participation in this research

| **Police force** | **Population (mid 2022 estimate)** | **Police recorded drug offences, year ending Dec 2023** | **Drug offence rate per 1,000** | **Participating in this research** |
| --- | --- | --- | --- | --- |
| Avon and Somerset | 1,765,400 | 2,640 | 1.50 | Yes |
| Bedfordshire | 715,900 | 1,821 | 2.54 | Yes |
| Cambridgeshire | 906,800 | 1,795 | 1.98 | No |
| Cheshire | 1,108,800 | 3,246 | 2.93 | No |
| Cleveland | 579,300 | 2,459 | 4.24 | Yes |
| Cumbria | 503,000 | 1,584 | 3.15 | Yes |
| Derbyshire | 1,067,000 | 2,311 | 2.17 | No |
| Devon and Cornwall | 1,810,400 | 3,976 | 2.20 | No |
| Dorset | 785,200 | 1,016 | 1.29 | No |
| Durham | 637,600 | 1,447 | 2.27 | Yes |
| Essex | 1,877,300 | 5,649 | 3.01 | No |
| Gloucestershire | 652,400 | 1,089 | 1.67 | No |
| Greater Manchester | 2,911,700 | 14,031 | 4.82 | Yes |
| Hampshire | 2,018,700 | 5,936 | 2.94 | Yes |
| Hertfordshire | 1,204,600 | 2,135 | 1.77 | No |
| Humberside | 943,000 | 1,596 | 1.69 | Yes |
| Kent | 1,875,900 | 5,271 | 2.81 | No |
| Lancashire | 1,550,500 | 2,862 | 1.85 | No |
| Leicestershire | 1,136,700 | 3,381 | 2.97 | No |
| Lincolnshire | 775,500 | 1,601 | 2.06 | No |
| Merseyside | 1,442,100 | 11,364 | 7.88 | Yes |
| Metropolitan | 8,855,300 | 38,918 | 4.39 | No |
| Norfolk | 925,300 | 1,952 | 2.11 | Yes |
| North Yorkshire | 828,100 | 1,430 | 1.73 | No |
| Northamptonshire | 792,400 | 2,287 | 2.89 | Yes |
| Northumbria | 1,466,200 | 3,350 | 2.28 | No |
| Nottinghamshire | 1,163,300 | 4,061 | 3.49 | No |
| South Yorkshire | 1,392,100 | 4,656 | 3.34 | No |
| Staffordshire | 1,146,200 | 1,926 | 1.68 | No |
| Suffolk | 768,600 | 1,419 | 1.85 | Yes |
| Surrey | 1,214,500 | 2,462 | 2.03 | No |
| Sussex | 1,721,000 | 3,928 | 2.28 | No |
| Thames Valley | 2,549,700 | 6,008 | 2.36 | Yes |
| Warwickshire | 607,600 | 1,044 | 1.72 | No |
| West Mercia | 1,314,000 | 2,193 | 1.67 | No |
| West Midlands | 2,953,800 | 7,717 | 2.61 | Yes |
| West Yorkshire | 2,378,100 | 8,780 | 3.69 | Yes |
| Wiltshire | 751,500 | 1,032 | 1.37 | No |
| **Total** | **57,095,500** | **170,373** | **2.98** | **38** |
| **Participating** | **22,791,400** | **72,836** | **3.20** | **15** |
| **Not participating** | **34,304,100** | **97,537** | **2.84** | **23** |

## Supplementary references

[1]	Ashley Mills, ‘Technical Note: Deterministic Linkage of Police Force Data and National Drug Treatment Monitoring Service Data’, Dec. 2025. [Online]. Available: https://kar.kent.ac.uk/112400/

[2]	Ministry of Justice, ‘A Technical Guide to Ethnicity and the Criminal Justice System, 2022’, HM Government, London, Jan. 2024. [Online]. Available: https://assets.publishing.service.gov.uk/media/65b1491bf2718c000dfb1cb2/A_Technical_Guide_to_Ethnicity_and_the_CJS_2022.pdf
